# Giant Helical Dichroism in Twisted Hollow-Core Photonic Crystal Fibers

**DOI:** 10.1021/acsphotonics.4c02019

**Published:** 2025-02-10

**Authors:** Christof Helfrich, Michael H. Frosz, Francesco Tani

**Affiliations:** † University of Erlangen-Nürnberg, Staudtstraße 7/B2, 91058 Erlangen, Germany; ‡ Max Planck Institute for the Science of Light, Staudtstraße 2, 91058 Erlangen, Germany; § University of Lille, CNRS, UMR 8523−PhLAM−Physique des Lasers Atomes et Molécules, Lille, F-59000, France

**Keywords:** waveguides, photonic crystal fiber, chiral, orbital angular momentum

## Abstract

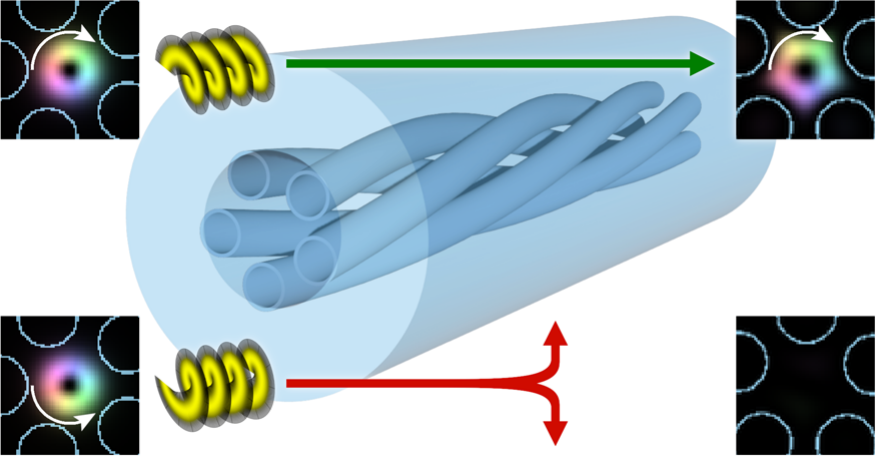

We show that twisted single-ring hollow-core fibers can exhibit strong helical dichroism, i.e., a different transmission depending on the orbital angular momentum of the launched light. Experimentally, we observe loss differences of at least 40 dB/m over a broad spectral range (>60 THz). We investigate the effect via analytical and numerical studies and show that considerably higher differential loss can be achieved over a broader spectral range (>180 THz). Our observation provides new routes for controlling the polarization state, extends previous studies of circularly dichroic waveguides, and has many potential applications, such as the realization of new polarizing elements in previously inaccessible spectral regions, chiral sensing, broadband generation of vortex beams, and optical communication.

## Introduction

Chiroptical effects provide a convenient route to optically differentiate between the two enantiomer forms of chiral media and control the polarization of light. The well-established circular dichroism (CD) has been exploited for a long time to characterize chemical and biological samples and to realize optical elements for selecting the helicity of circularly polarized light, also known as spin angular momentum (SAM).^1^ The recent advances in the understanding and fabrication of nanostructures, metamaterials, and fibers have led to new optics with tailored, broadband, and enhanced chiral optical properties and platforms to enhance enantioselectivity.^2−8^

In comparison, helical dichroism (HD), the analogue of CD based on orbital angular momentum (OAM), is much less studied and has started attracting attention only in the last two decades. Nevertheless, the effect has been already observed in a wide range of systems, both natural (e.g., molecules^9,10^) and artificial,^11^ and a few works have also reported its use for distinguishing point-like (with respect to the light wavelength) enantiomers with opposite chirality.^12,13^ Set off against CD, defining OAM dichroism is less straightforward as different combinations of SAM and topological charge (i.e., OAM value) can lead to different responses.^14^ In the past few years, a growing number of groups have reported nanostructures and metasurfaces exhibiting HD and achieving large extinction ratios (between ≈1 dB and ≈6 dB for topological charges >4) by designing them with characteristic dimensions comparable to (and larger than) the wavelength of light.^15−17^ On the other hand, helically dichroic waveguides have not been reported yet. This is despite the possibility of designing them for strong CD and excellent guidance of OAM-carrying modes and providing a promising and alternative platform for shaping the properties of light and enhancing the interaction of chiral light with matter.^18−20^

Here, we report a photonics platform with unprecedently strong and broadband helical dichroism (HD) in the visible spectral range. By using a 25 cm-long twisted single-ring hollow-core photonic crystal fiber (SR HC-PCF), we measured a loss difference of at least 10 dB over a spectral range spanning more than 60 THz (≈ 180 THz according to numerical simulations). We discuss the measurements, and with the aid of a simple analytical model supported by finite element modeling, we investigate the dichroism origin and how it depends on the fiber design and the twist rate. We designed and fabricated the fiber to guide in the visible when filled with typical solvents (e.g., water, ethanol, acetonitrile) so that it can be used to study chiral solutions and exhibit HD after filling it with water. Additionally, the effect can be observed with gas filling by adjusting the twist rate.

## Methods

**Figure 1:**
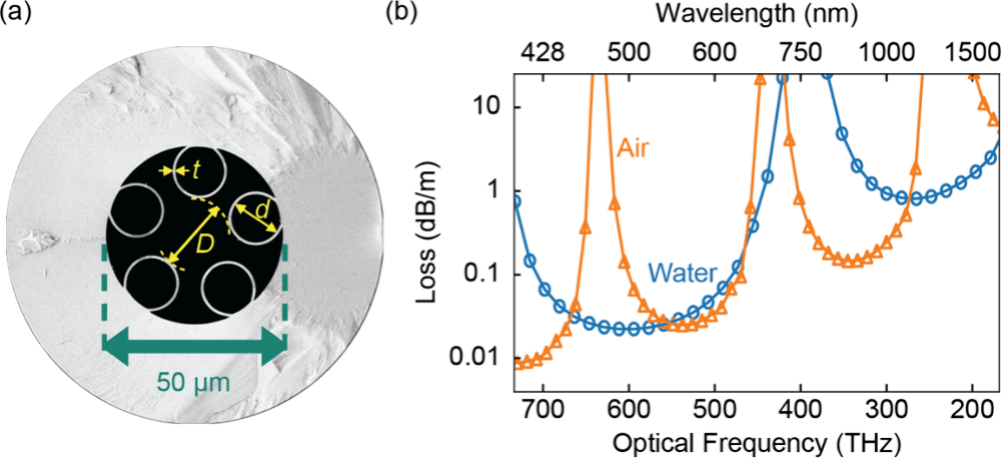
(a) End facet of the twisted HC-PCF with a glass jacket (SEM image); (b) Calculated confinement loss of the fundamental mode when the fiber is filled with water (blue round markers) or air (orange triangles) and linear interpolation (lines).

In the experiment, we use a twisted SR HC-PCF whose scanning electron micrograph (SEM) is shown in Figure 1a. The waveguide has a core of diameter *D* = 20.3 μm, which is surrounded by five glass capillaries of diameter *d* = 13.8 μm and of thickness *t* = 670 nm, and is twisted along its length at a rate α = 157 rad/m. The selected geometry (i.e., core diameter and *d*/*D* = 0.68) ensures high loss for almost all of the higher order modes (HOMs)^21−23^ over the fiber length (25 cm), and for the chosen *t*, the fiber exhibits a low-loss transmission window spanning from ≈430 to ≈610 nm when filled with water. The transmission window is delimited by spectral anticrossings between the guided modes and capillary-wall resonances affecting both the loss and the dispersion of the waveguide and occurring at wavelengths
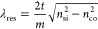
1where *m* is a positive integer number and *n*_si_ and *n*_co_ are the refractive indices of the silica glass and the fiber core.^24,25^ In Figure 1b we show the optical confinement loss calculated using finite element modeling (FEM) for the waveguide filled with water (*n*_co_ = *n*_water_ ≈ 1.335) and air (*n*_co_ = *n*_air_ ≈ 1).

**Figure 2:**
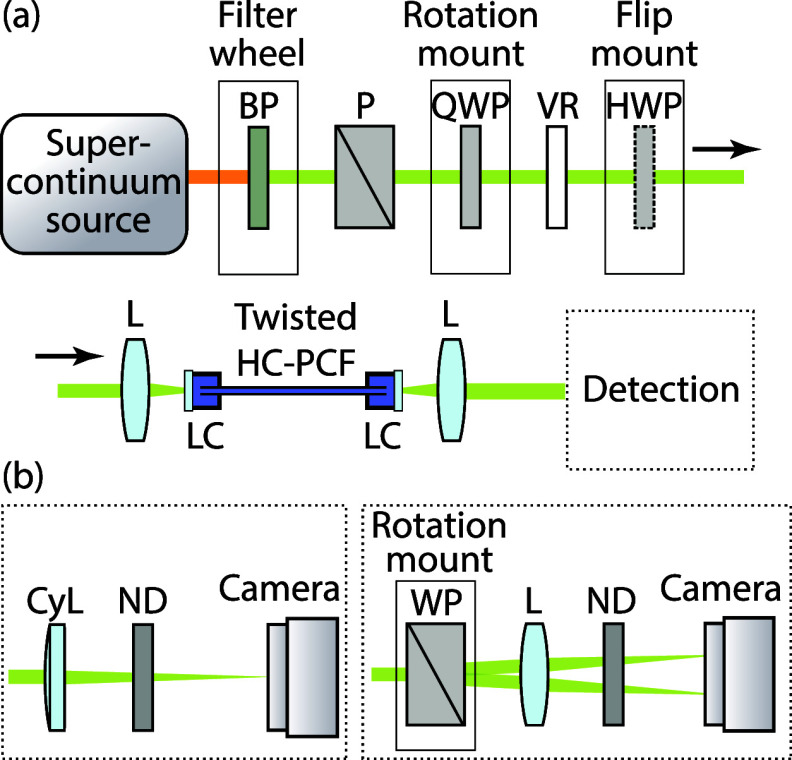
(a) Experimental setup and (b) two different configurations of the detection section (left: determination of the topological charge; right: imaging polarimeter): BP = bandpass filter, P = crystal polarizer, QWP = achromatic quarter-wave plate, VR = voltage-controlled LC vortex retarder, HWP = achromatic half-wave plate, L = achromatic lens, LC = liquid cell, CyL = cylindrical lens, ND = neutral density filter, WP = Wollaston prism.

To characterize the HC-PCF and measure its helical dichroism, we use the experimental setup depicted in Figure 2a. The HC-PCF end facets are placed into liquid cells with fused silica windows for in- and outcoupling of the light and filled with distilled water using a syringe with a microparticle filter. As a light source, we use a supercontinuum extending down to ≈500 nm obtained by pumping a tapered solid-core PCF with nanosecond pulses from a Q-switched laser (7 μJ/pulse, 1030 nm, 500 Hz).^26^ To control the topological charge  and the circular polarization (CP) handedness *s*, we use a combination of a quarter waveplate, a vortex retarder (ARCoptix Spiral Plate standard), and a half-wave plate. Preceding these optics, we use bandpass filters with a bandwidth of 10 nm at full-width half-maximum (FWHM) and centered at 520, 550, and 580 nm to select a narrow portion of the light spectrum. The retardance of the vortex retarder is then tuned for each wavelength by adjusting the driver voltage (ARCoptix LC driver). With the vortex retarder tuned to a phase shift of π, we generate four different vortex beams by combination of topological charge  = ±1 and circular polarization (CP) handedness *s* = ±1. After selecting the sign of  by rotating the quarter-wave plate (fast axis at ±45° with respect to the polarizer P), we change the sign of *s* by moving the half-wave plate in or out of the beam path. For transmission measurements of the fundamental mode ( = 0), we adjust the driver voltage of the vortex retarder for a phase shift of 2π.

We launch the light into the water-filled HC-PCF via an achromatic lens (5 cm focal length), and after collimating the output with a second lens (3 cm focal length), we use two different detection configurations, which are shown in Figure 2b. For the determination of , we use a cylindrical lens (30 cm focal length)^27−29^ followed by a neutral density filter (ND) so as to maximize the dynamic range, and a beam profiler (Figure 2b, left configuration).

For an accurate characterization of the guided light, we use the right detection configuration in Figure 2b to obtain near-field maps of intensity and of the absolute value of the normalized *S*_3_ Stokes parameter at the end facet, by using the relation
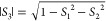
2where *S*_1_ and *S*_2_ are the other Stokes parameters.

## Results

**Figure 3:**
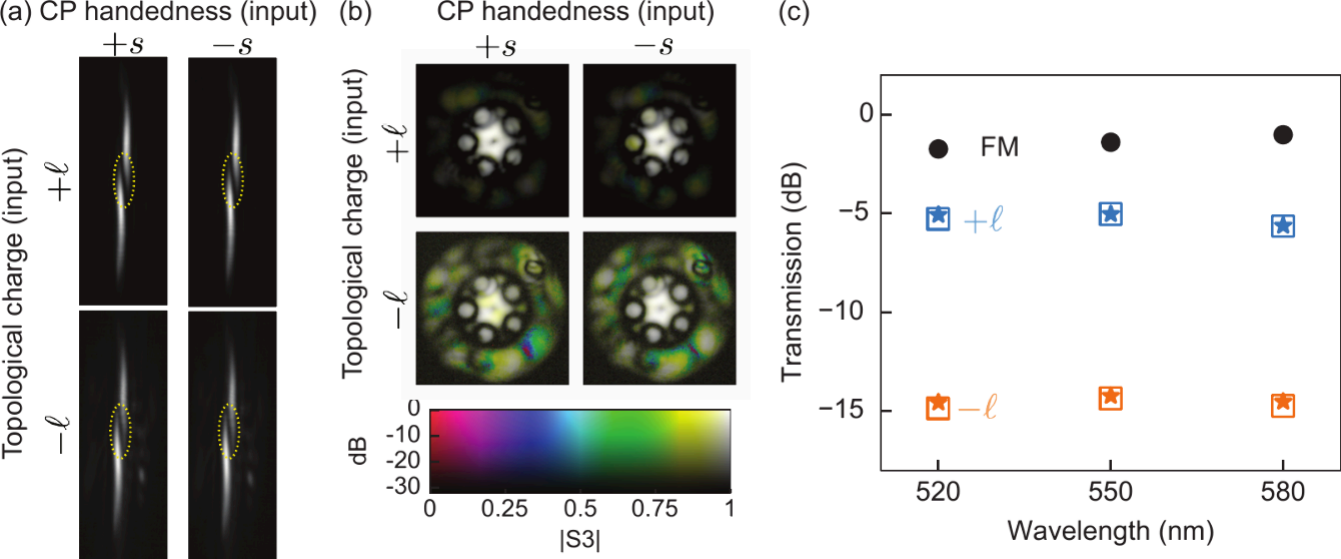
(a, b) Camera images characterizing the mode field at the end facet of the twisted HC-PCF. Launching light at 580 nm with  (top rows), the transmission is ten times larger than for  (bottom rows): (a) Intensity patterns at the focus of a cylindrical lens (the dashed yellow lines highlight the zeros), (b) near-field mapping of relative intensity and |*S*_3_| (each image is normalized for visibility). (c) Measured transmission for excitation of the fundamental mode (FM,  = 0) and for vortex beams coupled in the twisted HC-PCF (square markers for “+*s*”, stars for “–*s*”), twist rate α = 157 rad/m, fiber length = 25 cm.

In Figure 3, we show the characterization of the fiber output for different combinations of topological charge and handedness of the input light.

We use the images in Figure 3a for the determination of  (as described in ref (27)). These are captured using light at 580 nm and using the cylindrical lens to focus the fiber output on the camera. In this fashion, we identify a topological charge of magnitude one corresponding to the single central zero highlighted in the images–with the same sign for all the recorded images and wavelength (we present camera images only for 580 nm). An opposite sign of the topological charge would appear as a mirror image; however, this is not the case for any image. The observation and the much lower transmission of the -light indicate that the confinement loss depends on the sign of the topological charge.

The unvarying sign of  at the output, despite changing its sign at the input, can be explained in two ways: (i) By coupling from a high-loss vortex mode (labeled as “”) into a weakly attenuated vortex mode (labeled as “”) due to nonzero integral overlap, and (ii) by impurities of the in-coupled light with respect to the sign of .

In Figure 3b, we show the obtained near-field maps of |*S*_3_| using the 580 nm filter. The maps reveal that the fraction of light in the surrounding glass jacket (see Figure 1a) amounts to 4.2% in the  case and 32% in the  case. As the fiber jacket acts as a non-polarization-maintaining, highly multimode waveguide, any light coupled into it reaches the end-facet in a highly mixed polarization state. Thus, in our analysis, we disregard the light in the jacket to avoid introducing systematic measurement errors. In this fashion, we obtain a mean value of |*S*_3_|, weighted by intensity and averaged across the liquid-filled area (50 μm diameter, see Figure 1a), larger than 0.99 in the weakly attenuated case and larger than 0.97 in the high-loss case.

The fiber transmission for each  and *s* of the input light can now be measured with a laser power meter (Ophir Nova II/PD300-UV, averaging 10 s). In Figure 3c, we show the transmission values obtained after accounting for the Fresnel reflection of the optical windows, and subtracting the fraction of light in the jacket.

The measurements reveal that the HC-PCF exhibits strong and broadband helical dichroism: For each of the three wavelengths, the transmission of the vortex modes launched into the fiber varies by at least 10 dB depending on the sign of 

At the end-facet, the highly attenuated -mode is masked by a weak -mode, affecting the estimation of the attenuation difference from the measured transmission (as discussed below, simulations predict a much larger attenuation difference).

As opposed to the work reporting circular dichroism in twisted HC-PCFs,^5^ we do not observe any significant dependence of the transmission on the sign of *s*. Here, we use a much lower twist rate, and we observe broadband HD and conservation of *s* along the fiber length.

## Discussion

To gain insight into the origin of the observed helical dichroism, we calculate, via FEM in a helicoidal coordinate system,^30^ the complex propagation constant of the four lowest-order vortex modes of a twisted HC-PCF filled with water. For this, we use a fiber geometry based on the SEM image in Figure 1a and vary the capillary diameter *d* and the twist rate while keeping the original core diameter *D*. It is worth noticing that unlike step-index fibers, which support guided modes, SR HC-PCFs support only leaky modes. The optical loss experienced by these can be exceptionally low^31^ and can be enhanced (or suppressed) for a selected set of modes by adjusting the fiber geometry.

**Figure 4:**
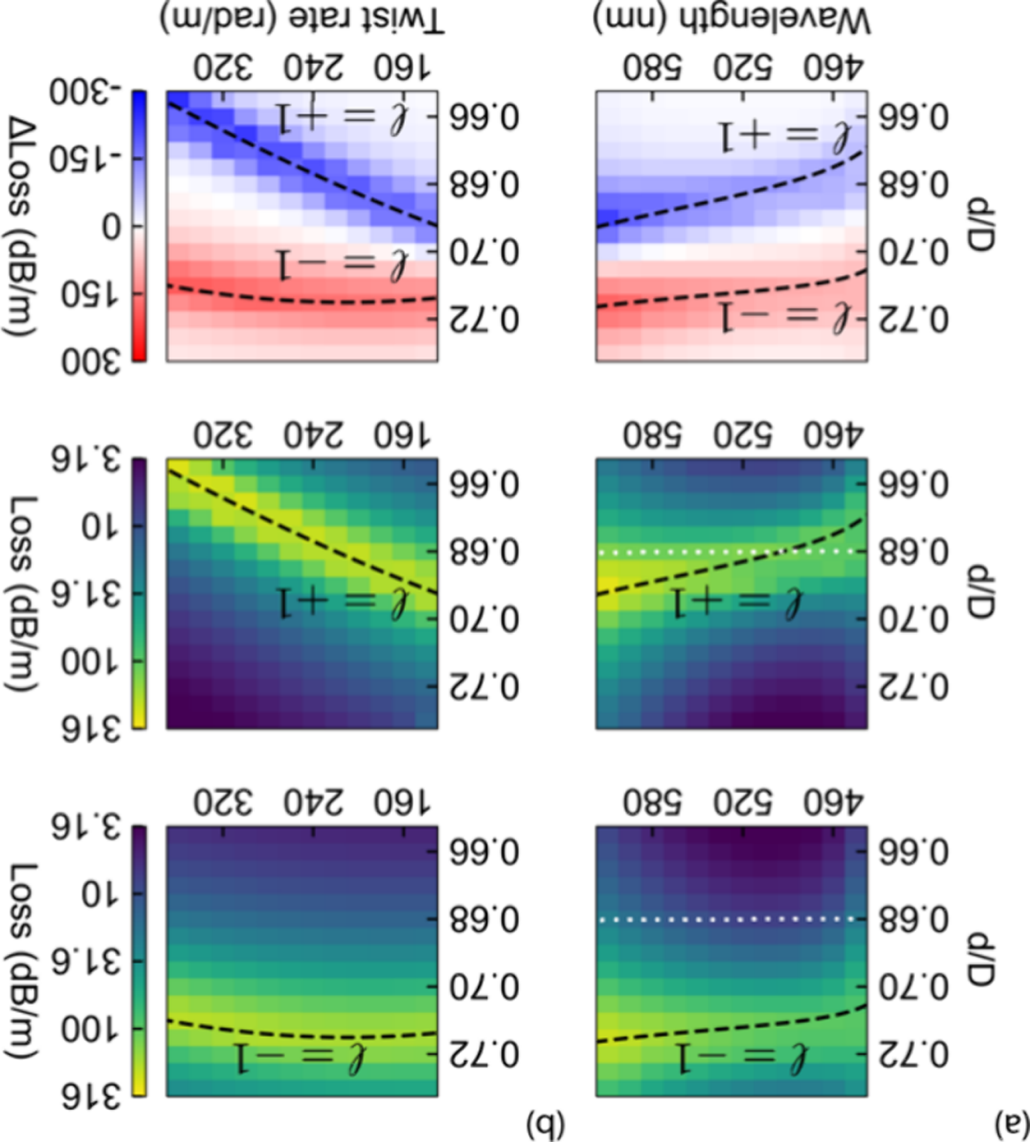
Influence of the ratio *d*/*D* of capillary diameter *d* to core diameter *D* on the confinement loss of vortex modes with  = −1 (top),  = +1 (middle) and loss difference (bottom) calculated by FEM simulations for *D* = 20.3 μm; dashed lines: Analytical phase matching condition eq 3, white dotted lines: Fabricated fiber parameter value *d*/*D* = 0.68, (a) wavelength dependence at constant twist rate of 157 rad/m, (b) twist rate dependence at constant wavelength of 580 nm.

In Figure 4 we show the calculated confinement loss of the vortex modes with  = ±1 as a function of the ratio *d*/*D*, (a) wavelength (with the twist rate fixed at 157 rad/m), and (b) twist rate (with the wavelength fixed at 580 nm). With the white-dotted line in the first two plots of Figure 4a, we highlight the calculated confinement loss of the fabricated fiber. The simulation results predict strong and broadband HD slightly above and slightly below the value of *d*/*D* = 0.70.

The experimental and numerical results can be understood in the laboratory coordinate system in terms of phase matching between vortex modes of the central hollow core with the highly lossy fundamental modes of the surrounding capillary tubes. To describe this, we use the following expression:^22,32^
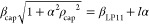
3where β_LP11_ and β_cap_ are the propagation constants of an idealized LP_11_ core mode and LP_01_ capillary tube mode of an untwisted fiber, α is the twist rate in rad/m, and ρ_cap_ = (*d* + *D*)/2 is the radial distance of the capillary tube centers from the fiber axis. The square root term on the left-hand side of eq 3 accounts for the increase in the path length of a capillary tube due to the twist. The second term on the right-hand side of eq 3 describes the azimuthal phase structure of the vortex mode with topological charge , observed along the helical path around the central phase singularity.

We estimate β_LP11_ and β_cap_ via the extended Marcatili model^33−35^ for hybrid modes of a capillary tube and using the effective core diameter^21^*D*_eff,LP11_ = *f*_LP11_*D* (and *d*_eff_ = *d*). We determine the scaling factor *f*_LP11_ by fitting the expression for the real part of the mode index to the FEM simulation results (transformed back to the laboratory coordinate system) far away from resonant coupling between core and capillary tube modes (i.e., for *d*/*D* ratios where the phase matching in eq 3 is satisfied) and in the limit α → 0. In this fashion, we find the linear approximation
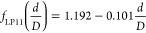
4

As shown in Figure 4a,b, the numerical solutions of eq 3 coincide with the high loss region obtained via FEM, thus indicating that eq 3 can be used to design helically dichroic HC-PCFs. Furthermore, the excellent agreement with FEM simulations shows that additional correction terms for the propagation constant of vortex modes such as twist-induced circular birefringence (on the order of 1 rad/m for  = 1, according to our FEM simulations), nondegeneracy with respect to the sign of  (on the order of 0.1α), or increased optical path length for higher-order core modes,^22^ are not necessary to explain the observed strong and broadband HD.

It is worth noticing that when the HC-PCF shown in Figure 1a is filled with air, we do not observe strong HD in the experiment. This is due to the inverse proportionality between core refractive index *n*_co_ and the required α to achieve phase matching, which can be seen after first order approximations of eq 3 (details are given in the SI). The reciprocal relation suggests that a faster twist rate is required to observe HD in an air-filled fiber. This is confirmed by preliminary FEM simulations (not shown) revealing that HC-PCFs can be designed so as to exhibit strong and broadband HD also when filled with air.

## Conclusion

We have shown through experiments, simulations, and analytic considerations that a twisted HC-PCF can be designed for strong and broadband HD, providing attenuation ratios exceeding 10 dB. Our numerical simulations show that the twisted HC-PCFs can exhibit optical losses differing by over 100 dB/m for modes with opposite topological charges. Thus, increasing the fiber length can lead to an enormous HD. However, measuring it requires suppressing any coupling to the weakly attenuated vortex mode, which may result from imperfect in-coupling or cross-talk along the fiber length.

We believe that the results of this work represent a significant step forward for the ongoing development of high-discrimination-power polarizing elements in hard-to-reach spectral regions and of hollow-core waveguides with advanced polarization properties. Usually, the complex beating patterns between modes make the analysis of the polarization properties of multimode waveguides a difficult task. This is greatly simplified in a waveguide that supports two instead of four weakly attenuated vortex modes of order 1, in addition to two low-loss fundamental modes. We foresee a huge potential for many applications, such as chiral sensing (e.g., of pharmaceuticals in liquid solutions), optical communication, and generation of broadband vortex beams.

## Supporting Information Available

Additional experimental details and references (PDF)
