# Supplementary material for: Giant Helical Dichroism in Twisted Hollow-Core Photonic Crystal Fibers
Source: ACS Photonics. 2025 Feb 10;12(2):564–9. doi: 10.1021/acsphotonics.4c02019 (PMC11844252; doi:10.1021/acsphotonics.4c02019)
Supplement: Supplementary file 1 — ph4c02019_si_001.pdf [file ph4c02019_si_001.pdf]

Supporting Information for

Giant Helical Dichroism in Twisted Hollow-Core Photonic Crystal Fibers

Christof Helfrich[1,2], Michael H. Frosz[2], Francesco Tani[2,3]

[1]*University of Erlangen-Nürnberg, Staudtstraße 7/B2, 91058 Erlangen, Germany*
[2]*Max Planck Institute for the Science of Light, Staudtstraße 2, 91058 Erlangen, Germany*
[3]*University of Lille, CNRS, UMR 8523—PhLAM—Physique des Lasers Atomes et Molécules, Lille, F-59000, France*

Here, we show that the realization of helically dichroic single-ring hollow-core photonic crystal fibers (SR HC-PCFs) requires faster twist rates when the waveguide is filled with lower refractive index media. As mentioned in the main text, the critical twist rate to achieve phase matching is inversely proportional to the core medium refractive index n_{co}, and the relation can be derived via first-order approximations of the phase matching condition[1,2] given by Eq. (3) in the main text:

$$\beta_{\mathrm{cap}}\sqrt{1+\alpha_{cr}^2{\rho_{\mathrm{cap}}}^2} = \beta_{\mathrm{LP11}} + \ell\alpha_{cr}\,, \tag{3}$$

where β_{LP11} and β_{cap} are the propagation constants of an idealized LP_{11} core mode and LP_{01} capillary tube mode of an untwisted fiber, α_{cr} is the critical twist rate in rad/m, $\ell = \pm 1$ is the topological charge of the vortex mode, $\rho_{\mathrm{cap}} = (d+D)/2$ is the radial distance of the capillary tube centers from the fiber axis, D is the core diameter, and d is the capillary diameter.

For this, we use a first-order expansion of β_{cap} and β_{LP11} given by the capillary model[3–5] (however, higher order terms are necessary to achieve the good agreement with the FEM simulation results shown in Fig. 4):

$$\beta_{\mathrm{cap}} \approx n_{co}k_0 - \frac{1}{2}\frac{1}{n_{co}k_0}\frac{{u_{\mathrm{cap}}}^2}{(d/2)^2}\,, \tag{S1}$$

$$\beta_{\mathrm{LP11}} \approx n_{co}k_0 - \frac{1}{2}\frac{1}{n_{co}k_0}\frac{{u_{\mathrm{LP11}}}^2}{(f_{\mathrm{LP11}}D/2)^2}\,, \tag{S2}$$

where $k_0 = 2\pi/\lambda$ is the vacuum wavenumber, n_{co} is the core medium refractive index, $u_{\mathrm{cap}} \approx 2.405$, $u_{\mathrm{LP11}} \approx 3.8317$ are the first zeros of the Bessel functions J_0, J_1, and f_{LP11} is a scaling factor without unit[6]. By applying a first-order approximation to the square root term, we rewrite Eq. (3) as

$$\beta_{\mathrm{cap}} + \beta_{\mathrm{cap}}\frac{1}{2}\alpha_{cr}^2{\rho_{\mathrm{cap}}}^2 = \beta_{\mathrm{LP11}} + \ell\alpha_{cr}. \tag{S3}$$

We simplify the second term on the left-hand side of Eq. (S3) by setting $\beta_{\text{cap}} \approx n_{co} k_0$ (this is valid for $d \gg \lambda$), before we insert (S1) and (S2) into (S3). After basic algebraic transformations, we find the result

$$n_{co}{}^2 k_0{}^2 D^4 \alpha_{cr}^2 - n_{co} k_0 D^2 \alpha_{cr} \, \frac{8\ell}{(d/D+1)^2} + \frac{16}{(d/D+1)^2} \left(\frac{u_{\text{LP11}}{}^2}{f_{\text{LP11}}{}^2} - \frac{u_{\text{cap}}{}^2}{(d/D)^2} \right) = 0\,, \quad \text{(S4)}$$

which can be rewritten as the quadratic equation

$$x^2 - x\,B + C = 0\,, \quad \text{(S5)}$$

by the substitution $x := n_{co} k_0 D^2 \alpha_{cr}$. The comparison between (S4) and (S5) reveals, that the terms B and C depend only on the parameter d/D, except that B also depends on ℓ. After a solution x of Eq. (S5) has been found, the required twist rate to achieve phase matching is given by

$$\alpha_{cr} = \frac{\lambda}{2\pi\, n_{co} D^2}\, x\,. \quad \text{(S6)}$$

The reciprocal relation between n_{co} and α_{cr} is evident in Eq. (S6), which suggests that a faster twist rate is required to observe HD in an air-filled fiber. This is confirmed by preliminary FEM simulations (not shown) revealing that HC-PCFs can be designed so as to exhibit strong and broadband HD also when filled with air.

References

(1) Edavalath, N. N.; Günendi, M. C.; Beravat, R.; Wong, G. K. L.; Frosz, M. H.; Ménard, J.-M.; Russell, P. St.J. Higher-Order Mode Suppression in Twisted Single-Ring Hollow-Core Photonic Crystal Fibers. *Opt. Lett.* **2017**, *42* (11), 2074–2077. https://doi.org/10.1364/OL.42.002074.

(2) Ma, X.; Liu, C.-H.; Chang, G.; Galvanauskas, A. Angular-Momentum Coupled Optical Waves in Chirally-Coupled-Core Fibers. *Opt. Express* **2011**, *19* (27), 26515–26528. https://doi.org/10.1364/OE.19.026515.

(3) Finger, M. A.; Joly, N. Y.; Weiss, T.; Russell, P. St.J. Accuracy of the Capillary Approximation for Gas-Filled Kagomé-Style Photonic Crystal Fibers. *Opt. Lett.* **2014**, *39* (4), 821–824. https://doi.org/10.1364/OL.39.000821.

(4) Marcatili, E. a. J.; Schmeltzer, R. A. Hollow Metallic and Dielectric Waveguides for Long Distance Optical Transmission and Lasers. *Bell Syst. Tech. J.* **1964**, *43* (4), 1783–1809. https://doi.org/10.1002/j.1538-7305.1964.tb04108.x.

(5) Zeisberger, M.; Schmidt, M. A. Analytic Model for the Complex Effective Index of the Leaky Modes of Tube-Type Anti-Resonant Hollow Core Fibers. *Sci. Rep.* **2017**, *7* (1), 11761. https://doi.org/10.1038/s41598-017-12234-5.

(6) Uebel, P.; Günendi, M. C.; Frosz, M. H.; Ahmed, G.; Edavalath, N. N.; Ménard, J.-M.; Russell, P. St.J. Broadband Robustly Single-Mode Hollow-Core PCF by Resonant Filtering of Higher-Order Modes. *Opt. Lett.* **2016**, *41* (9), 1961–1964. https://doi.org/10.1364/OL.41.001961.
